# An Incidental Finding of an Indigo-Blue Liver in a Patient With Dubin-Johnson Syndrome Confirmed via Genetic Testing

**DOI:** 10.7759/cureus.79505

**Published:** 2025-02-23

**Authors:** Macarena Viñuela, Camila Sotomayor, Emilio Morales, Miguel Pérez, Javiera Torres, Francisco Barrera, Jorge A Martínez

**Affiliations:** 1 Otolaryngology, Pontificia Universidad Católica de Chile, Santiago, CHL; 2 Hepatobiliary and Pancreatic Surgery, Pontificia Universidad Católica de Chile, Santiago, CHL; 3 Pathology, Pontificia Universidad Católica de Chile, Santiago, CHL; 4 Gastroenterology, Pontificia Universidad Católica de Chile, Santiago, CHL

**Keywords:** abcc2 gene mutation, blue liver syndrome, conjugated hyperbilirubinemia, dubin-johnson syndrome, laparoscopic cholecystectomy

## Abstract

We present the case of a 29-year-old female with chronic jaundice and a history of dyshidrosis, previously treated with cyclosporine. She also had hypertriglyceridemia managed with fibrates and was referred for evaluation of gallstone disease. Imaging confirmed gallstones without bile duct abnormalities. Laboratory tests revealed conjugated hyperbilirubinemia (total bilirubin: 3.0 mg/dL, direct bilirubin: 2.95 mg/dL). During laparoscopic cholecystectomy, a striking blue liver appearance was observed. A liver biopsy confirmed Dubin-Johnson syndrome (DJS). Genetic analysis revealed two pathogenic ABCC2 variants: c.2077G>A (p.Gly693Arg) and a novel mutation, c.513del (p.Tyr172Thrfs*6). This report highlights a rare "blue liver" presentation of DJS, thereby expanding the differential diagnosis of blue liver syndrome.

## Introduction

Dubin-Johnson syndrome (DJS) is a rare autosomal recessive disorder characterized by chronic conjugated hyperbilirubinemia and distinct pigment deposition in hepatocytes. First described by Dubin and Johnson in 1954 [1], the syndrome is defined by its unique clinicopathological features, including persistent jaundice and a liver with dark pigmentation due to impaired bilirubin excretion. This impairment results from mutations in the ABCC2 gene, which encodes the canalicular multispecific organic anion transporter 2 (MRP2/cMOAT), essential for bilirubin glucuronide transport into bile [1].

Advances in molecular genetics have provided significant insights into the pathogenesis of DJS. Mutations within the ATP-binding cassette region of ABCC2 disrupt transporter function, leading to intracellular bilirubin accumulation [2]. Wada et al. identified specific mutations in this gene that directly impair MRP2 activity [3], while Wu et al. demonstrated that recurrent mutations, such as p.G693R, are associated with severe phenotypic presentations in DJS patients [4]. Notably, the molecular understanding of ABCC2 mutations has been pivotal in differentiating DJS from other cholestatic conditions such as progressive familial intrahepatic cholestasis and Rotor syndrome.

Clinically, DJS is considered benign, with patients often asymptomatic apart from intermittent jaundice. However, rare presentations, such as the striking "brilliant blue liver" described by Bong et al. [5], challenge the conventional perception of the disease. These atypical cases underscore the phenotypic variability of DJS and its potential overlap with other hepatobiliary disorders, such as sinusoidal obstruction syndrome. This paper discusses a novel case of DJS with an unusual intraoperative finding of a "blue liver," alongside genetic confirmation of ABCC2 mutations, further expanding the clinical and molecular spectrum of this rare disorder.

## Case presentation

**Figure 1:**
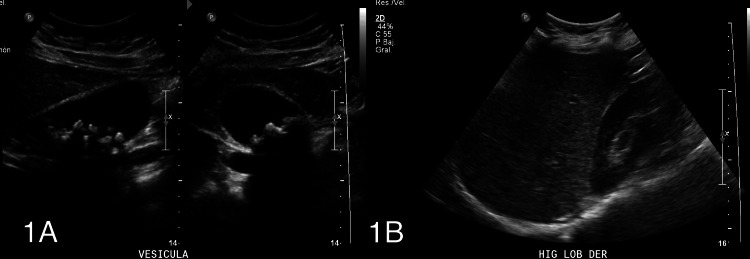
Abdominal ultrasound A: Gallbladder is distended, with thin walls and multiple mobile echogenic images casting posterior acoustic shadows, compatible with gallstones. No intrahepatic or extrahepatic bile duct dilation is observed in this imaging modality. B: Liver of normal size and morphological characteristics, with a slight increase in echogenicity, possibly indicative of mild steatosis. No evidence of focal lesions with an aggressive appearance within its parenchyma

**Figure 2:**
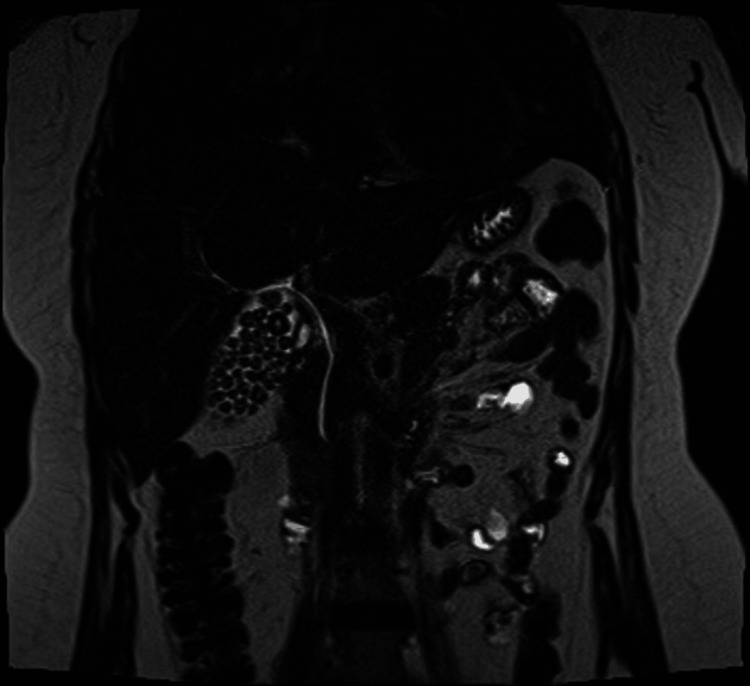
MRI: coronal plane The gallbladder is distended, measuring 9.7 cm in its longest axis, with numerous faceted calculi in its lumen ranging in size from 5 to 11 mm. The cystic duct exhibits normal caliber and insertion. The intrahepatic bile ducts display normal caliber and signal intensity. The extrahepatic bile duct measures 4 mm in internal diameter at the level of the common hepatic duct. No hypointense filling defects suggestive of choledocholithiasis are observed in the common bile duct MRI: magnetic resonance imaging

**Figure 3:**
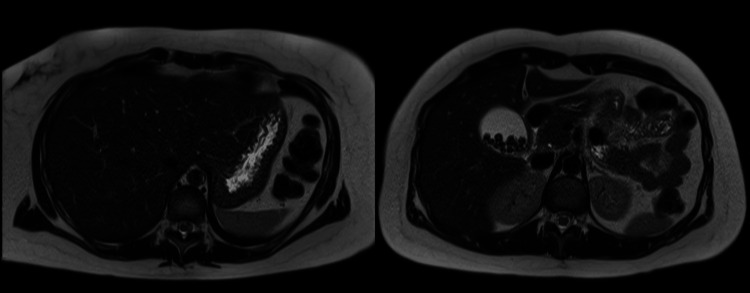
MRI: axial plane MRI: magnetic resonance imaging

**Figure 4:**
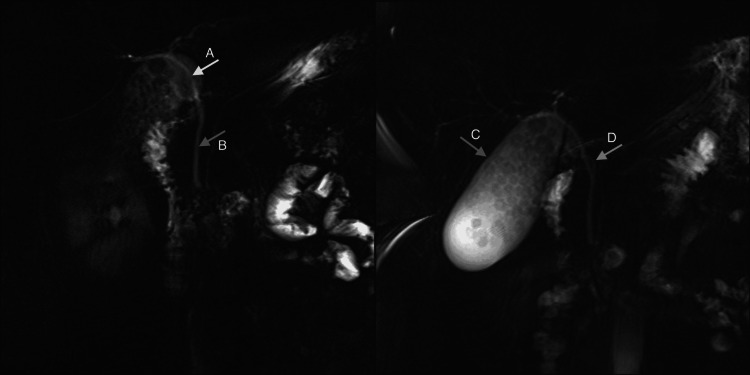
MRC: radial bile duct reconstruction Arrow A: Common hepatic duct: 4 mm in diameter. Arrow B: Common bile duct: No hypointense filling defects are observed in the common bile duct, suggestive of stones. Arrow C: Gallbladder: distended. 9.7 cm in length along its long axis, with numerous faceted calculi in its lumen, ranging in size from 5 to 11 mm. Arrow D: Cystic duct: normal caliber and insertion MRC: magnetic resonance cholangiography

A 29-year-old female, with chronic fluctuating jaundice since childhood and a past medical history of severe dyshidrosis treated with cyclosporine and receiving fibrates for hypertriglyceridemia, was referred for surgery assessment for symptomatic gallstone disease. An ultrasound (Figures 1A, 1B) and MRI cholangiography (Figures 2, 3, 4) confirmed gallstones with normal bile ducts and no choledocholithiasis.

The patient's laboratory tests showed conjugated hyperbilirubinemia (total bilirubin: 3.0 mg/dL, direct bilirubin: 2.95 mg/dL), with otherwise normal liver tests and cell blood count (Table 1).

**Table 1:** Preoperative laboratory tests ALT: alanine aminotransferase; AST: aspartate transaminase; GGT: Gamma-glutamyl transferase; INR: international normalized ratio; sGOT: serum glutamic-oxaloacetic transaminase; sGPT: serum glutamic pyruvic transaminase

Test	Result	Reference range
sGOT (AST)	15 U/L	<25 U/L
sGPT (ALT)	16 U/L	<30 U/L
GGT	20 U/L	<40 U/L
Alkaline phosphatase (FA)	59 U/L	30–100 U/L
Total bilirubin	3.0 mg/dL	<1.0 mg/dL
Direct bilirubin	2.95 mg/dL	<0.3 mg/dL
Prothrombin time	88%	70–120%
INR	1.1	
Serum creatinine	0.6 mg/dL	0.5–0.9 mg/dL
Hemoglobin	12.6 g/dL	12–16 g/dL
White blood cells	9100/uL	4500–11000/uL
Platelets	276000/uL	140000–400000/uL

**Figure 5:**
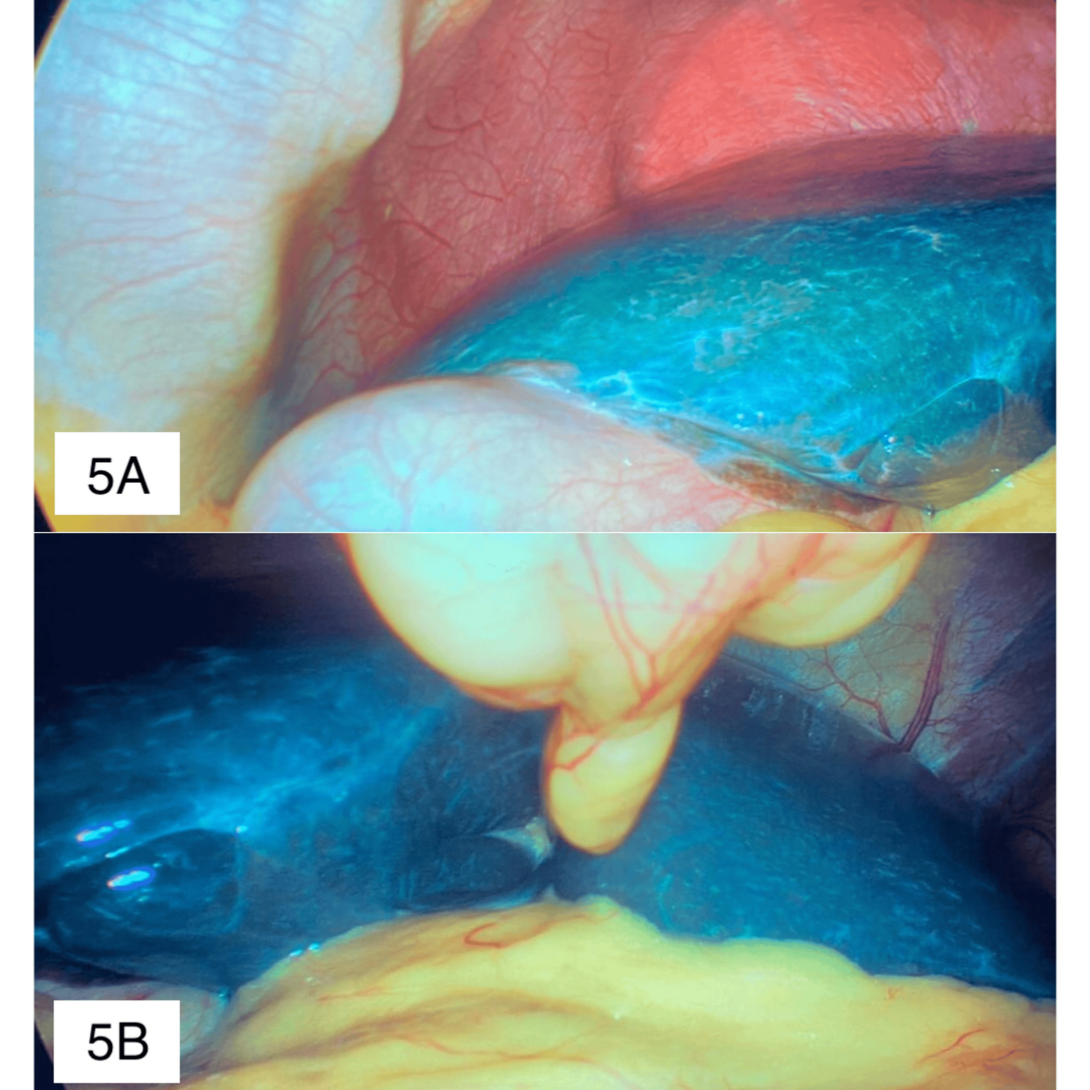
Intraoperative laparoscopic view during cholecystectomy Remarkable blue discoloration of the liver can be seen, with normal consistency and smooth edges

The patient underwent laparoscopic cholecystectomy, demonstrating a remarkably blue liver appearance intraoperatively, with normal consistency and smooth edges (Figures 5A, 5B).

**Figure 6:**
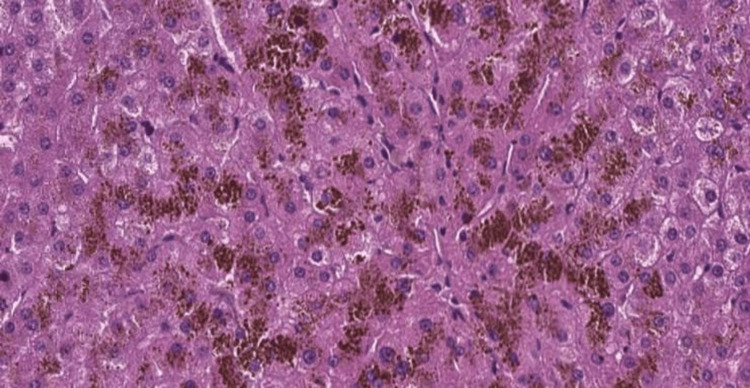
Biopsy findings - 1 Microscopic examination of hepatic tissue with marked cytoplasmic dark-brown granular pigment in hematoxylin and eosin preparations

**Figure 7:**
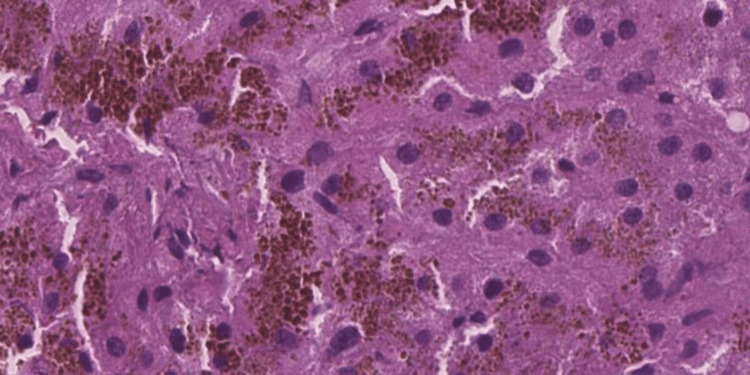
Biopsy findings - 2 Microscopic examination of hepatic tissue with marked cytoplasmic dark-brown granular pigment in hematoxylin and eosin preparations

**Figure 8:**
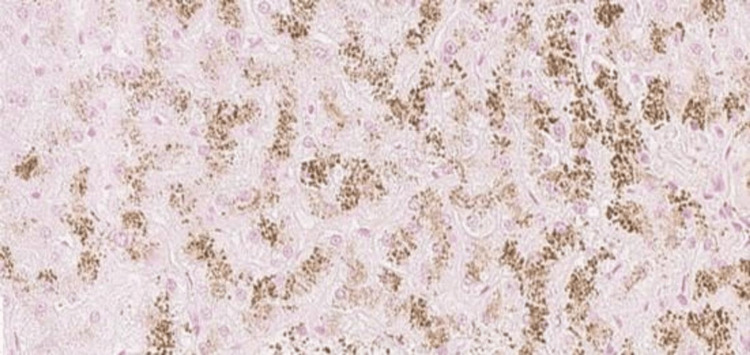
Biopsy findings - 3 Prussian blue stain shows ochre yellow granular pigments in the cytoplasm of hepatocytes

Liver biopsy showed an accumulation of granular brown, refringent pigment in the apical side of the hepatocyte with no other remarkable findings, characteristic of DJS (Figures 6, 7, 8).

A complete sequence deletion/duplication analysis of the 136 genes listed in the Invitae Cholestasis Panel (Invitae 1400 16th Street, San Francisco, CA) was conducted, demonstrating two pathogenic variants in the ABCC2 gene (NM_000392.4). The missense variant c.2077G>A(p.Gly693Arg) has been already reported [1] in individuals with DJs, and regarding the second variant c.513del(p.Tyr172Thrfs*6), a loss of function mutation has not been previously reported in DJS. The parents were not available for the segregation study.

## Discussion

DJS is a rare autosomal recessive hereditary disorder with an estimated prevalence of one in 100,000 inhabitants [1,4]. It is characterized by a genetic mutation in the canalicular multispecific organic anion transporter (MRP2/cMOAT), encoded by the ABCC2 gene. This mutation impairs bilirubin excretion, leading to chronic direct hyperbilirubinemia, long-standing jaundice, and the characteristic deposition of dark pigment in hepatocytes [2,6].

The accumulation of pigment in hepatocytes results in the hallmark characteristic of DJS: a liver with a blackened appearance upon gross examination. However, recent case reports have described an alternative presentation characterized by an intense, brilliant blue liver, expanding the phenotypic spectrum of the syndrome [6]. This variant, although rare, should be considered in the differential diagnosis of blue liver syndrome, a term traditionally associated with oxaliplatin-induced sinusoidal obstruction syndrome (SOS) [7]. Given the potential for clinical confusion, recognizing this novel presentation of DJS is crucial for appropriate diagnosis and management. In our patient, we identified a novel ABCC2 variant (c.513del), which expands the molecular spectrum of this condition. This raises an important question: does possessing this variant predispose patients to presenting with the "blue liver" phenotype rather than the classic "black liver"? This possibility certainly warrants further investigation.

Histologically, the liver of DJS patients exhibits a distinctive brown-black pigment deposition within centrilobular hepatocytes, particularly around the central veins. Under light microscopy, the liver architecture appears normal, but the pigment accumulates as dark, granular deposits, a feature not observed in Rotor syndrome. Electron microscopy reveals that the pigment is localized within lysosomes. Histochemical staining and physicochemical analysis suggest that this pigment is a melanin-like polymer derived from defective bilirubin metabolism [3,8]. Despite the marked liver discoloration, DJS is generally considered a benign condition, as liver function tests (aside from direct bilirubin elevation) remain within normal limits, and affected individuals do not develop progressive liver disease [9].

Molecular studies have elucidated the underlying pathophysiology of DJS. The ABCC2 gene encodes the MRP2 transporter, which plays a pivotal role in the biliary excretion of conjugated bilirubin and other organic anions. Mutations in ABCC2 disrupt transporter function, leading to intracellular accumulation of bilirubin-derived pigments [10]. Genetic testing for ABCC2 mutations can aid in confirming the diagnosis, particularly in atypical presentations [11]. Differentiating DJS from other causes of direct hyperbilirubinemia remains a critical aspect of clinical assessment. Table 2 summarizes how genetic testing can aid in this differentiation.

**Table 2:** Genetic differential diagnosis

Disease	Mutation
Dubin-Johnson syndrome (DJS)	Genetic mutation in the canalicular multispecific organic anion transporter (MRP2/cMOAT), encoded by the ABCC2 gene
Rotor syndrome	Autosomal recessive disorder caused by homozygous mutations in the SLCO1B1 and SLCO1B3 genes on chromosome 12; these genes encode the organic anion-transporting polypeptides OATP1B1 and OATP1B3, respectively
Progressive familial intrahepatic cholestasis (PFIC)	
PFIC1	ATP8B1 gene
PFIC2	ABCB11 gene (located on chromosome 2)
PFIC3	ABCB4 gene (located on chromosome 7)
Crigler-Najjar syndrome	Mutations in the UGT1A1 gene, located on chromosome 2, which encodes bilirubin uridine diphosphate-glucuronosyltransferase (B-UGT)
Gilbert-Meulengracht syndrome	Mutation in the promoter region of the UGT1A1 gene (2q37), leading to reduced activity of the uridine diphosphate-glucuronosyltransferase (UGT) enzyme
Wilson's disease	Mutations in the ATP7B gene, located on the long arm (q) of chromosome 13 (13q14.3); this gene encodes the ATPase 2 protein

Management of DJS remains primarily supportive, focusing on patient education and reassurance. Given its benign nature, no specific pharmacologic interventions are required, and the condition does not predispose patients to cirrhosis or hepatocellular carcinoma [12]. However, differentiating DJS from other causes of direct hyperbilirubinemia - such as rotor syndrome, progressive familial intrahepatic cholestasis, and hepatocellular dysfunction - remains a critical aspect of clinical assessment [13,14].

While DJS classically manifests with a black liver, the emerging recognition of a brilliant blue variant highlights the importance of expanding the differential diagnosis of blue liver syndrome. Further research is needed to understand the pathophysiological aspects of this rare presentation and its potential clinical implications.

## Conclusions

This report highlights a rare presentation of DJS in a 29-year-old woman with chronic jaundice and symptomatic cholelithiasis. The intraoperative discovery of a "blue liver," combined with characteristic histological findings and genetic confirmation of ABCC2 mutations, reinforces the phenotypic variability of DJS. The identification of a novel ABCC2 variant (c.513del) expands the molecular spectrum of this condition. Although DJS is a benign disease that does not progress to fibrosis or cirrhosis and does not require treatment, its diagnosis is crucial to rule out other hepatobiliary disorders that may cause liver damage and identify those that may be potentially treatable. While it classically presents as a "black liver", we discussed a rare case of a blue liver observed intraoperatively. Awareness of the blue liver phenotype is essential for differential diagnosis, particularly in cases of unexplained liver discoloration. This report underscores the importance of integrating clinical, radiological, histological, and genetic data for accurate diagnosis and management.

## References

[1] Dubin IN, Johnson FB (1954). Chronic idiopathic jaundice with unidentified pigment in liver cells; a new clinicopathologic entity with a report of 12 cases. Medicine (Baltimore).

[2] Arrese JM (1999). Identification of molecular defects in liver diseases: recent examples (Article in Spanish). Rev Med Chil.

[3] Toh S, Wada M, Uchiumi T (1999). Genomic structure of the canalicular multispecific organic anion-transporter gene (MRP2/cMOAT) and mutations in the ATP-binding-cassette region in Dubin-Johnson syndrome. Am J Hum Genet.

[4] Wu L, Li Y, Song Y (2020). A recurrent ABCC2 p.G693R mutation resulting in loss of function of MRP2 and hyperbilirubinemia in Dubin-Johnson syndrome in China. Orphanet J Rare Dis.

[5] Bong SH, Soon GS, Huang DQ (2021). A brilliant blue liver. Clin Gastroenterol Hepatol.

[6] Bosia JD, D'Ascenzo MV, Borzi S, Cozzi S, Defelitto JR, Curciarello JO (2008). The Dubin-Johnson syndrome: case report and review of literature (Article in Spanish). Acta Gastroenterol Latinoam.

[7] Rubbia-Brandt L, Lauwers GY, Wang H (2010). Sinusoidal obstruction syndrome and nodular regenerative hyperplasia are frequent oxaliplatin-associated liver lesions and partially prevented by bevacizumab in patients with hepatic colorectal metastasis. Histopathology.

[8] Baba N, Ruppert RD (1972). The Dubin-Johnson syndrome: electron microscopic observation of hepatic pigment--a case study. Am J Clin Pathol.

[9] Erlinger S, Arias IM, Dhumeaux D (2014). Inherited disorders of bilirubin transport and conjugation: new insights into molecular mechanisms and consequences. Gastroenterology.

[10] Fardel O, Jigorel E, Le Vee M, Payen L (2005). Physiological, pharmacological and clinical features of the multidrug resistance protein 2. Biomed Pharmacother.

[11] Wu L, Zhang W, Jia S (2018). Mutation analysis of the ABCC2 gene in Chinese patients with Dubin-Johnson syndrome. Exp Ther Med.

[12] Talaga ZJ, Vaidya PN (2023). Dubin-Johnson Syndrome. StatPearls [Internet]. Treasure Island (FL): StatPearls Publishing.

[13] Corpechot C, Barbu V, Chazouillères O (2020). Genetic contribution of ABCC2 to Dubin-Johnson syndrome and inherited cholestatic disorders. Liver Int.

[14] Tripathi N, Jialal I (2023). Conjugated Hyperbilirubinemia. StatPearls [Internet]. Treasure Island (FL): StatPearls Publishing.

